# Human resource development for a community-based health extension program: a case study from Ethiopia

**DOI:** 10.1186/1478-4491-11-39

**Published:** 2013-08-20

**Authors:** Hailay D Teklehaimanot, Awash Teklehaimanot

**Affiliations:** 1 Center for National Health Development in Ethiopia, Columbia University, Bole Sub City, Kebele 06, H No 447, PO Box 664, Code 1250, Addis Ababa, Ethiopia; 2 The Earth Institute, Columbia University, 475 Riverside Drive, Suite 401, New York, NY 10025, USA

**Keywords:** Health extension program, Health extension workers, Human resource, Infrastructure, Decentralized management, Primary care, Preventive and promotive health services, Service delivery strategy, Ethiopia

## Abstract

**Introduction:**

Ethiopia is one of the sub-Saharan countries most affected by high disease burden, aggravated by a shortage and imbalance of human resources, geographical distance, and socioeconomic factors. In 2004, the government introduced the Health Extension Program (HEP), a primary care delivery strategy, to address the challenges and achieve the World Health Organization Millennium Development Goals (MDGs) within a context of limited resources.

**Case description:**

The health system was reformed to create a platform for integration and institutionalization of the HEP with appropriate human capacity, infrastructure, and management structures. Human resources were developed through training of female health workers recruited from their prospective villages, designed to limit the high staff turnover and address gender, social and cultural factors in order to provide services acceptable to each community. The service delivery modalities include household, community and health facility care. Thus, the most basic health post infrastructure, designed to rapidly and cost-effectively scale up HEP, was built in each village. In line with the country’s decentralized management system, the HEP service delivery is under the jurisdiction of the district authorities.

**Discussion and evaluation:**

The nationwide implementation of HEP progressed in line with its target goals. In all, 40 training institutions were established, and over 30,000 Health Extension Workers have been trained and deployed to approximately 15,000 villages. The potential health service coverage reached 92.1% in 2011, up from 64% in 2004. While most health indicators have improved, performance in skilled delivery and postnatal care has not been satisfactory. While HEP is considered the most important institutional framework for achieving the health MDGs in Ethiopia, quality of service, utilization rate, access and referral linkage to emergency obstetric care, management, and evaluation of the program are the key challenges that need immediate attention.

**Conclusions:**

This article describes the strategies, human resource developments, service delivery modalities, progress in service coverage, and the challenges in the implementation of the HEP. The Ethiopian approach of revitalization of primary care through innovative, locally appropriate and acceptable strategies will provide important lessons to other poorly resourced countries. It is hoped that the approaches and strategies described in this paper will aid in that process.

## Background

Ethiopia is one of the sub-Saharan countries most affected by high disease burden reflected by the high rates of maternal and child mortality [1]. Poor nutritional status, infections, and high fertility rates, accompanied by low levels of access to essential health services, contribute to the high mortality rates seen in the country. In 1997, the Ethiopian government established a 20-year Health Sector Development Program (HSDP) to meet these challenges [2]. The HSDP is a comprehensive national plan and framework, implemented in four 5-year rolling plans. Under the first 5-year rolling plan of the HSDP, the overall performance of the health sector had improved; however, the ability to deliver essential services in rural settings was less successful [3]. In 2003, at the end of the first 5-year HSDP plan, only one-quarter of pregnant women received antenatal care, only one in ten births was attended by skilled personnel, and about one-third (32.7%) of children were fully immunized as the potential health service coverage was 61.3% [4]. As a result, the overall levels of disease burden, and child and maternal mortality appeared to have hardly shifted during the initial HSDP plan period [4], demonstrating that the standard health system through the HSDP model could not address the poor health situation of rural people.

Shortages and imbalances of human resources for health, geographical distance from health facilities, and socioeconomic factors aggravated by the poor health-seeking behaviors of the population were among the major obstacles to attaining wider access to health services [3]. Over 80% of the population in Ethiopia reside in remote rural localities, situated in a very scattered way, presenting a major challenge in the expansion of health facilities to the entire population. The limited capacity of the country in the production of paraprofessionals such as nurses and primary healthcare workers, as well as the challenges in retention of health workers in remote areas, lead to shortages and imbalances in the health workforce [5-7]. Ethiopia, like many developing countries, has suffered from ‘urban bias’, which keeps health professionals away from rural and remote areas, resulting in disparities of access to health services and health outcomes [5]. Beyond the lack of access to health services, the rural population is vulnerable to illnesses and particularly affected by widespread poverty and lack of access to clean water and sanitation facilities, which contribute to high disease burden [4].

As a response to these challenges, the Federal Ministry of Health (FMOH) sought a primary care strategy that could immediately be scaled up to address the major challenges in the health system, meet the health needs of the people and achieve the World Health Organization Millennium Development Goals (MDGs) within a context of limited country resources [8]. The Health Extension Program (HEP), which is a strategy for the scaling up of an institutionalized package of basic and essential promotive, preventive and limited curative health services, was introduced in 2004 as one of the primary components of the second 5-year HSDP plan [3]. The HEP service package comprises evidence-based interventions selected to address the major maternal, neonatal and child health problems as well as the major infectious and communicable diseases [8-14] that constitute approximately 60 to 80% of the health problems in the country [2]. The HEP service is delivered at health facility and community levels. The health facility care is delivered from the most basic health facility (health post), and is supported by health centers through referral linkage. To cover all rural villages with the HEP, over 15,000 health posts have been constructed, and over 30,000 female Health Extension Workers (HEWs) have been trained and deployed [4].

This article describes the strategies and components of the HEP, the human resource development process, the service delivery modalities, and the progress in service coverage in relation to the scale up of HEP. The challenges facing HEP are also outlined together with key recommendations for the path forward. The present work aims to increase the understanding of what is required for the successful scale up of primary care in poorly resourced countries, and the model described will provide potential approaches and strategies for the scale up of the recently launched One Million Community Health Workers (CHWs) Campaign [11].

## Case description

### Strategic approach

The federal government structure of Ethiopia is composed of nine National Regional States and two City Administrations, each with a Regional Health Bureau (RHB). The regions are further divided into 805 woredas (districts), each with a District Health Office (DHO). The 805 districts are further divided into about 15,000 kebeles (villages), each with an average of 5,000 inhabitants. The kebele is the lowest administrative government unit, where the HEP service delivery is organized. The design of the HEP reconciled the need for immediate response with the need for sustainability. The requirement for sustainability necessitated the design of an institutionalized and integrated health program with properly developed human capacity, infrastructures, decentralized management and political commitment [15-17]. The need for immediate response necessitated the design of cost-effective primary care that can be delivered from the most basic health facility and community level by primary health workers trained in a relatively short period at an affordable cost to the country [16,18]. The health service package as well as the strategies designed to institutionalize the HEP are described briefly below.

#### Primary health care unit (PHCU) infrastructure

To create a platform for the institutionalization of HEP, the healthcare delivery system in the country was reorganized into a four-tier system: Primary Health Care Units (PHCUs), District Hospitals, Zonal Hospitals and Specialized Hospitals. The PHCU, which comprises a cluster of 5 health posts and a referral health center, serves as the HEP implementation institutional framework for 25,000 people. The infrastructure development primarily focused on the construction of health posts in each kebele of 5,000 people and expansion of referral health centers. Thus, a comprehensive network of about 3,300 PHCUs was required to cover approximately 75 million people, which entailed the construction of over 15,000 health posts and 3,300 health centers. A health post has two or three multipurpose rooms for provision of integrated health services, designed for efficient use of resources [16]. The referral health centers were designed with adequate space for outpatient and inpatient care, as well as under-5 and maternal care (including rooms for basic emergency obstetric care). Through a multiphase approach, the health centers are being upgraded to provide comprehensive emergency obstetric care (C-EMOC), which requires an operating theater with a blood bank and means of transport.

The FMOH developed standard lists of equipment, drugs and supplies for equipping the health posts based on the HEP service package [17]. Key medical equipment includes examination beds, stethoscope, blood pressure apparatus, weighing scale, foetoscope, delivery bed and kit, neonatal resuscitation mask and bag, measuring board and tape, refrigerator, vaccine carriers, ice box, and first aid kit. The following are among the essential drug list for health posts: coartem, amoxicillin, oral rehydration salt, vitamin A, Iron and folic acid, ergometrine, oral misoprostol, oral contraceptive and Depo-Provera injections, and vaccines. In addition, health posts are equipped with furniture, medical supplies (disinfectants, rapid diagnostic test (RDTs), condoms, HIV test kits), guidelines and standard protocols, registers, posters and charts.

#### Comprehensive package of health intervention

The HEP service package comprises a range of evidence-basedhigh-impact and cost-effective interventions targeting the major health problems in the country [2,17]. The services were selected on their suitability for delivery at household, outreach and health facility levels [12]. Although the HEP service package includes limited basic curative services, it mainly focuses on promotional and preventive interventions designed to tackle cultural issues, develop personal and social skills, and increase health awareness that enables individuals to take action to promote their own health [8,12]. The promotion and prevention premise of the HEP reduces the need for curative and rehabilitative care, and assures greater efficiency of services in the form of time saved in consultation and reduced healthcare expenditure, which removes socioeconomic factors as a barrier to receiving needed care, leading to more equitable health outcomes and higher user satisfaction [8,19].

The HEP package includes 17 essential health services under 4 major program areas of care (Table 1): (1) family health, (2) disease prevention and control, (3) hygiene and environmental sanitation, and (4) health education and communication. The family health program, which includes family planning, antenatal care, assisted delivery, postnatal care, newborn care, immunizations, nutrition, deworming, and treatment of childhood illness, deals with maternal, newborn and child health (MNCH) to address the high maternal and child mortality [10,12,13]. The treatment of childhood illness through Integrated Community Case Management targets the leading causes of death in children: malaria, pneumonia, and diarrhea. The referral health centers provide emergency obstetric care, which is a key strategy in achieving the MDG on maternal health [10,13]. The disease prevention and control program includes promotive and preventive interventions to combat the major infectious diseases: malaria (diagnosis and treatment of cases, promotion of use and distribution of nets), HIV/AIDS (education on prevention, counseling and testing, distribution of condoms), and tuberculosis (sputum collection, identification of defaulters) [9,20]. The interventions under hygiene and environmental sanitation include promotion of proper handling of water supplies (at the source and home), basic sanitation (proper disposal of excreta, solid and liquid wastes), personal and food hygiene, and healthy home environment (such as ventilation, separate kitchen and animal place) [14], which contribute to reduction in child mortality, maternal health and infectious diseases [9]. The specific activities of HEWs include educating households on health and environment benefits of the various interventions, as well as demonstration, supervision (inspection) and even assisting households (in manual labor) in construction of necessary infrastructures such as latrines, garbage disposal pits and improved cooking stoves. Any capital costs (mainly labor and local materials) incurred for infrastructure development for some of the hygiene and environmental sanitation interventions are the responsibility of the households.

**Table 1:** Health service package included under the health extension program (HEP)

**Major program areas**	**Health service package**
Family health services	Maternal and child health
	Family planning
	Immunization
	Adolescent reproductive health
	Nutrition
Disease prevention and control	HIV/AIDS
	Tuberculosis
	Malaria
	First aid
Hygiene and environmental sanitation	Excreta disposal system
	Solid and liquid waste management
	Water supply safety measures
	Food hygiene
	Healthy home environment
	Arthropods and rodent control
	Personal hygiene
Education and communication	Cross cutting

#### Health human resources

One of the distinctive strategies in the design of HEP is the use of female health workers (except in pastoralist areas) recruited from respective communities. This approach was designed to provide HEP services in ways acceptable and appropriate to all people, in a country that has more than 80 ethnic groups, languages and cultures. It was employed to limit the high turnover of health personnel and to address the gender, social and cultural factors that affect the expectations and the behavior of the health service providers and users [5,21]. The use of female health workers is particularly important to empower women in making healthy decisions for themselves and their families, since malnutrition, low birthweight, early childhood morbidity and micronutrient deficiencies, and maternal mortality are closely intertwined with the status of women in the community [22,23]. Two female Health Extension Workers are stationed in each health post. The HEWs are formally employed and salaried by the respective districts with the aim of preventing attrition and ensure sustainability [6,7]. Thus, more than 30,000 HEWs are needed to staff all health posts. Voluntary health promoters also support the work of HEWs, although they are not considered as staff of the health posts. The referral health centers are staffed with health officers, nurses, laboratory technicians, and other professionals who can provide curative services and basic emergency obstetric care (B-EMOC). When the health centers are upgraded with an operation theater for C-EMOC, professionals with emergency obstetric care and surgical skills are also deployed.

#### Management and health information system

Following the 1993 policy of decentralization, which provided administrative context with the aim of devolving power to district authorities, decision-making processes are shared between the different levels of the health system. The FMOH and RHBs are responsible for policy formulation and regulation, resource acquisition, pre-service training, and technical support. Management and coordination of HEP service delivery, by contrast, falls under the jurisdiction of the district, which is important for the success of the program [15]. The district oversees the construction of the PHCU facilities and recruitment of HEW candidates. The DHO allocates and manages resources, and along with the referral health centers, provide logistical and technical support during planning, implementation, monitoring, and evaluation of HEP. The DHO is staffed with the required management and supervisory professional staff. HEW supervisors, who are trained and equipped with the necessary logistics, provide supportive supervision, which is essential for quality improvement and job satisfaction [24-26]. In addition to creating a referral linkage, the PHCU creates a structure for close technical and logistical support to the health posts from the health centers. At the kebele level, HEWs are accountable to the kebele council, which is responsible for administrative support for the HEP. One of the two HEWs is a member of the council, and prepares work plans with the involvement of the council and community to ensure that the plans focus on local health problems.

The FMOH has reformed the health management information system (HMIS) to produce timely information for planning management and decision making. The number of reporting formats has been reduced, and HMIS units and database have been established at all levels of the health system. However, the remote location of the health posts and paper-based record-keeping procedures present a challenge for reporting the HEP information. This can lead to incomplete data and time lags between reporting and use of data for decision making, which hinders optimal health system performance. This has implications for monitoring and responding to epidemiological changes, operating a demand-driven supply chain, planning tailored interventions and evaluating health worker performance. Currently, the FMOH is seeking a mechanism to record, transmit, aggregate, and analyze real-time critical HEP data, and the alternative options described in the One Million Community Health Workers report based on the experiences in sub-Saharan Africa could be used by the FMOH in selecting the optimal technology for the HEP [11].

#### Political and partner commitment, and community engagement

The government is committed to provide basic healthcare to the entire population. The full implementation of HEP services at village level costs US$5.1 per capita per year, which is comparable to the US$5.63 per capita per year estimated for the CHW subsystem [18]. The estimated cost does not include any infrastructure development costs incurred by households for the implementation of the hygiene and environmental sanitation program. The village level HEP and expansion of first referral health centers with functional B-EMOC costs US$7.5 per capita per year. The cost increases to US$13.3 per capita with functional C-EMOC and clinical care, which is much lower than the recommended cost of a primary healthcare system [27,28]. The share of the estimated cost for HEP with C-EMOC by program component is as follows: health service delivery and quality of care, 17.7%; new construction, expansion and transport, 20.6%; human resource development, 4.9%; strengthening pharmaceuticals, 32.3%; IEC, 2.6%; management, Health Management Information System (HMIS) and monitoring and evaluation, 21.8%; and healthcare financing, 0.1%.

Financial resources for HSDP, including the HEP, come from the government treasury and development partners. The HEP was formulated in coordination with other government sectors (such as education and agriculture) and Health Development Partners to make full use of internal and external resources. The government recognizes that donor-supported programs, which are usually short-term and short-lived successes, have sometimes distorted and deformed health systems leading to fragmentation and duplication of efforts. The FMOH and donors worked together to revise their approach and pool resources to improve coordination and harmonization in order to achieve longer-term sustainable programs. The major objective was to have the ‘one plan, one budget and one report’ approach at all levels of the health system. A Code of Conduct instrument between the FMOH and its major partners was signed in 2005 to guide everyone’s actions in support of HSDP. The community also contributed local materials and labor for the construction of health posts; however, since all services at the health post are free of charge, out-of-pocket spending does not contribute to health expenditure at the health post level. The partnership with the Federal Ministry of Education was important for the training of the required human resources. Despite the high commitment of the government and partners to increase the total per capita health expenditure from about US$6 in 2004 to around US$16 in 2010 [4], there is a significant financial gap to scale up HEP as per the standard.

Community participation is one of the principles in the design of HEP to ensure ownership and empower the poor to have a more central role in the program [29,30]. At the initial stage, the community participated in infrastructure development and recruitment of health workers. The community also participates in planning, implementation, and monitoring of the HEP, ensuring that people are active partners in decision making about resources, defining priorities, and ensuring accountability [30].

#### Pastoralist health services

Pastoralist and nomadic peoples constitute about 10% of the country’s population. Nomadic and pastoral communities have many special health needs that are not completely met by the largely static health-post-based HEP that has been established for the agrarian communities. This gap prompted FMOH to establish an appropriate health service delivery for the pastoralist population. One of the major organizational transformations in the FMOH was creating the Pastoralist Health Promotion and Disease Prevention Directorate to focus attention on the health of the pastoralist populations. The 17 HEP packages were adapted to pastoralists’ needs. The FMOH designed modified PHCUs appropriate for pastoralist communities that focused on outreach health services. Moreover, due to a shortage of female candidates and cultural issues, and a shortage of high school graduates, sixth grade males were recruited and received 6 months of training to serve as HEWs.

### Implementation process

#### Human resource development process

Curriculum and teaching materials were developed for the 17 HEP service packages and other supportive and basic courses by a team of national and international experts. Nurses and environmental sanitation professionals were selected and trained to serve as instructors for HEW training. A selection committee comprised of community members and representatives from district health, capacity building and education offices, was responsible for the recruitment of HEW candidates. Recruitment criteria required that candidates be females who completed at least tenth grade education, were 18 years or older, residents of the kebele, and committed to returning to their respective kebele after completion of training.

A total of 40 Technical and Vocational Education Training Schools were established across the country by the Regional Education Bureaus, which was designed to facilitate accreditation and certification as part of the training. The FMOH and RHBs are responsible for follow-up and regulation of training quality. The HEW candidates received 1 year of intensive theoretical and practical training on the HEP service package, which is the same length of training as advocated by the One Million CHW Campaign [11,18]. Once the HEWs are deployed, the regulations on performance-based promotion, leave, absence, transfer, working time, and other conditions of work are based on the general civil service regulations. Among the ten regions in the country, seven regions initiated training of HEWs in 2004. The implementation of the HEP was delayed by 2 years in the predominantly nomadic and pastoralist regions of Afar, Gambella and Somali until the HEP packages were adapted to pastoralist needs.

To produce adequate nurses and health officers for the referral health centers, existing training institutions were expanded, and new training institutions under RHBs have been opened. A master level training of mid-level health workers on emergency surgery was started in five universities to mitigate the shortage of trained surgeons and provide C-EMOC services at referral health centers. This strategy was designed because it is expensive and time consuming to produce adequate numbers of trained surgeons. Moreover, the performance of mid-level health workers trained in basic surgery has been shown to be comparable to those of trained surgeons [31].

#### Service delivery modalities and referral

The service delivery strategies adopted for HEP includes health facility care (health post and referral health center), and community and household care. The HEWs spend 25% of their time at the health post and 75% of their time at the community and household level. Upon deployment, HEWs conduct a baseline assessment that is instrumental for planning purposes. All HEP services including the curative services are provided for free to remove financial barriers to care [19].

The health posts serve as an essential institutional framework for the implementation of the HEP, and HEWs establish contact between the community and the health system, bringing healthcare services to everyone. At the health post, HEWs provide family planning service, antenatal care, assisted normal delivery, postnatal care, newborn care, immunizations, growth monitoring, nutritional advice, HIV voluntary testing and counseling, first aid, basic curative service, and referral services. The linkage of health posts with referral health centers within the PHCUs creates a seamless care pathway for the community and brings curative care including emergency surgery services close to the community [10,32].

At community and household levels, HEWs implement promotional and preventive interventions to create appropriate healthy behaviors and to improve knowledge and attitude toward health-seeking behaviors. The interventions include promotion and provision of contraceptives, antenatal care including nutritional advice and micronutrient supplementation, clean delivery, basic newborn care, child nutrition (such as exclusive breastfeeding, complementary feeding, cooking nutritious meals, and vitamin A supplementation), immunization, use of bed nets, HIV prevention, sanitation, and hygiene (including support and supervision in the construction of latrines, disposal pits and healthful homes). The health promotion program is implemented through direct household visits by HEWs and the use of intermediate community structures including model families, women-centered health development armies, and school health program.

Model families are households that adopt and apply all 16 packages of the HEP services after receiving 96 hours of training on the service packages over 3 to 4 months. The training strategies include basic health action, persuasion, motivation, negotiation, encouragement, demonstration, provision of health services and transforming households into clean and safe home environments and maintaining a healthy life style. Households for model-family training are selected based on their involvement in other development work, and acceptance and credibility within the community. Model families are considered early adopters of desirable health practices and role models, and influence other households to adopt desired practices and behaviors through diffusion of health messages. The model-family households are organized as leaders of the one-to-five Health Development Army network, which is one of the innovations conceived to facilitate their efforts in community promotion activities. Each network comprises five women and a model family who are the leaders of the network. The one-to-five network serves as a forum for exchange of concerns, priorities, problems and decisions related to the health status of women, which was designed to empower women in particular and the family in general in health decision making leading to democratization of health and to community partnership. The networks are supported by the HEWs and are responsible for the preparation of plans and ensuring their completion, for the collection of health information, and also for conducting weekly meeting to review progress and submitting monthly reports.

Through collaboration with the education sector, thousands of schools across the country are involved in health promotion programs. Schools are regarded as an agent for change, and HEWs educate and encourage schoolteachers and students in order to address health and health-related issues at home, in the community and at school. Moreover, schoolchildren acquire long-lasting life skills, which allow them to lead healthier lifestyles. Other sectors such as agriculture are also involved and address the main factors of ill health and the burden of disease. The involvement and commitment of the agriculture sector contributes in reducing malnutrition and food insecurity through promotion of food supply and proper nutrition. Other community health promotion strategies include the use of community conversions, women and youth associations, and traditional associations.

In addition to the promotional and preventive interventions, HEWs provide integrated management of childhood illnesses at community and household level considering the low treatment-seeking behavior of the people. Moreover, HEWs identify defaulters, clients and patients that need closer attention and immediate referral. Other responsibilities include monitoring disease incidence and investigation of out breaks to take appropriate measures and prevent the spread of disease.

### Implementation status and outcome

**Figure 1:**
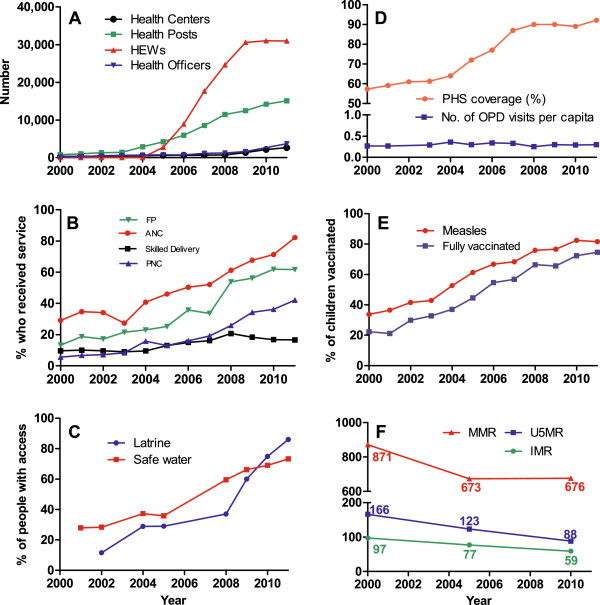
**Trend of health outcome indictors in relation to health extension program (HEP) expansion. (A)** Number of health facilities and personnel, **(B)** percentage of women who received maternal health services, **(C)** percentage of people who have access to safe water and latrine, **(D)** potential health service (PHS) coverage and outpatient (OPD) attendance per capita, **(E)** percentage of children vaccinated, and **(F)** maternal mortality ratio (MMR), infant mortality rate (IMR) and under-5 mortality rate (U5MR), 2000 to 2011.

Information on key performance indicators was extracted from the Health and Health Related Indicator report of the FMOH for 2000 to 2011 [4], which covers a period before and after the launch of the HEP. Health facility and administrative reports from all regions are used to calculate and publish the indicators in the annual reports. The indicators are not summary estimates of coverage values, but actual values calculated from sum of services received in all health facilities divided by respective national level denominators. Data on infrastructure (health posts and health centers), numbers of human resources (HEWs and health officers), numbers of women and children who received maternal and child health services, and numbers of outpatient visits for the period 2000 to 2011 are presented in Table 2. Data on child and maternal mortality was obtained from Demographic and Health Survey report [1]. The trend for some of the key performance indicators for the period 2000 to 2004 (pre-HEP period) was compared to the period 2004 to 2011 (post-HEP period). Figure 1 shows the trend for the performance indicators in relation to the level of human resource and infrastructure development. The trend for human resource and infrastructure development is presented in Figure 1A, and the targets have been achieved addressing the physical inaccessibility of health services in remote and scattered localities. About 33,819 HEWs were trained and deployed between 2005 and 2011, and the number of health posts increased from 2,899 in 2004 to 15,095 in 2011. The majority of the HEWs are female, completed 10 years of schooling, and are residents of the kebele as per the recruitment criteria. The health posts have been equipped with the necessary furniture and medical equipment. Although the target for constructing referral health centers has not been achieved, the number of operational health centers has increased by 413% from 519 in 2004 to 2,660 in 2011. Only one-third of these health centers provide B-EMOC services. The number of health officers trained and deployed to the health centers by the end of 2011 was 3,702. Over the same period, more than 3,000 HEW supervisors equipped with the necessary logistics have been deployed.

**Table 2:** Number of infrastructure and health workers, and maternal and child health services and outpatient visits in Ethiopia, 2000 to 2011

**Year**	**Population**	**Infrastructure**		**Human resources**		**No. of women who received:**				**No. of one year old children vaccinated**		**No. of outpatient (OPD) visits**
		**No. of health centers**	**No. of health posts**	**No. of HEWs **	**No. of health officers**	**Family planning (FP) service**	**Antenatal (ANC) service**	**Assisted skilled delivery**	**Postnatal (PNC) service**	**For measles**	**Fully**	
2000	63,495,000	356	833	0	201	1,667,386	815,518	165,446	151,029	904,567	585,528	17,143,650
2001	65,344,000	382	1,023	0	296	2,404,276	905,283	259,083	176,596	948,073	548,799	17,642,880
2002	67,220,000	412	1,311	0	484	2,289,959	914,935	258,387	190,899	988,471	711,368	12,189,204
2003	69,127,021	451	1,432	0	631	2,942,734	752,916	248,495	182,606	1,050,923	787,319	18,879,043
2004	71,066,000	519	2,899	0	683	3,223,182	1,150,134	266,349	446,672	1,320,804	927,531	25,405,141
2005	73,043,510	600	4,211	2,737	776	3,631,247	1,218,922	359,434	393,418	1,582,642	1,147,520	22,468,875
2006	75,067,000	635	5,955	8,901	715	5,420,461	1,407,574	421,483	433,887	1,861,831	1,387,936	24,620,248
2007	77,127,000	690	8,528	17,653	1,151	5,112,538	1,434,976	451,700	523,217	1,714,473	1,387,351	24,737,524
2008	79,221,000	732	11,446	24,571	1,242	8,010,630	1,724,268	589,011	727,702	1,959,682	1,699,081	18,835,927
2009	77,812,236	1,362	12,448	30,578	1,606	8,521,309	1,948,553	524,367	987,497	2,018,758	1,711,721	23,498,667
2010	79,894,802	2,142	14,192	30,995	-	9,956,168	2,113,669	497,328	1,071,435	2,220,525	1,947,263	23,134,941
2011	81,911,074	2,660	15,095	30,948	3,702	9,594,993	2,403,088	485,809	1,230,433	2,270,245	2,073,624	24,977,125

A piecewise linear function was used to capture the difference in slope before and after the launch of the HEP. Linear spline regression with a knot at year 2004 was fit for each indicator. The slope in each period represented the average change in coverage per year. The statistical significance of each slope as well as the difference in slopes between the two periods was tested (Table 3). With the scale up of HEP, the potential health service coverage, which only increased from 57.3% in 2000 to 64% in 2004, reached 92.1% in 2011. The outpatient attendance per capita did not improve despite improvement in potential health service coverage. Outpatient attendance is not expected to improve at the same rate as the expansion of HEP due to the premises of HEP where it only offers limited basic curative service; however, the availability of the limited curative service as well as improvement in treatment seeking and referral linkage should have contributed to the outpatient attendance.

**Table 3:** Trend of selected health indicators before and after Health Extension Program (HEP) was introduced into the health system in Ethiopia, 2000 to 2011

	**Linear spline: years 2000 to 2004**		**Linear spline: years 2004 to 2011**		**Difference between the two slopes**	
**Indicators**	**Coefficient (slope)**	***P *value**	**Coefficient (slope)**	***P *value**	**Change in slope**	***P *value**
Potential health service	2.96	0.02	4.11	0.000	1.16	0.45
Per capita attendance	0.02	0.046	−0.007	0.145	−0.02	0.05
Family planning	1.82	0.13	6.30	0.000	4.48	0.015
Antenatal care (ANC)	1.82	0.09	6.08	0.000	4.27	0.01
Skilled delivery	0.64	0.31	1.14	0.000	0.5	0.56
Postnatal care (PNC)	1.17	0.15	4.35	0.000	3.18	0.012
Measles	5.74	0.000	4.52	0.000	−1.22	0.33
Fully vaccinated	5.03	0.000	5.46	0.000	0.42	0.72
Latrine	2.1	0.34	8.7	0.000	6.63	0.045
Safe water	1.72	0.05	6.09	0.000	4.37	0.002

The investments in human resource development and infrastructure have resulted in a significant improvement of health service coverage indicators since the launch of the HEP (Table 3). Contraceptive acceptance rate increased significantly (*P* = 0.00) following the launch of HEP from 23% in 2004 to 61.7% in 2011, while the improvement was non-significant (*P* = 0.13) during the pre-HEP period. Similarly, the coverage of antenatal care, skilled delivery, and postnatal care improved significantly (*P* = 0.00) from 40.8%, 9.5%, and 15.8%, respectively in 2004 to 82.2%, 16.6%, and 42.1%, respectively in 2011, while there was no significant improvement during the pre-HEP period. The skilled delivery and postnatal care coverage, however, still remain low during the post-HEP period. The coverage of measles and fully vaccinated children in 2011 was 81.5% and 74.5%, respectively, which improved significantly during both pre-HEP and HEP periods. Hygiene and environmental sanitation indicators have also shown significant improvement after the launch of HEP. The percentage of people with access to improved latrine and safe water reached 86% and 73.3%, respectively in 2011 from 28.9% and 37.3%, respectively in 2004. The average annual improvement in family planning, antenatal care, postnatal care, improved latrine, and safe water coverage was significantly higher for the post-HEP compared to the pre-HEP period, while the average change in potential health service, skilled delivery, and immunization coverage per year was not significantly different between the two periods (Table 3). Although the pre-HEP and post-HEP slopes for potential health service and immunization coverage were not significantly different, the country has achieved high coverage in these indicators.

The increased access to health services and the promotion, availability and provision of contraceptive methods, antenatal care, and postnatal care at community level, and promotion of hygiene and sanitation services by HEWs, has contributed to the improvement in these indicators.

The under-5 mortality rate (U5MR) dropped from 166 in 2000 to 123 in 2005, and to 88 in 2011 per 1,000 live births, and infant mortality rate (IMR) from 97 in 2000 to 77 in 2005, and to 59 per 1,000 live births in 2011. The reduction in child mortality is explained by the improvement in child health indicators and availability of treatment service for the leading causes of death in children: malaria, pneumonia, and diarrhea [9,10]. The maternal mortality ratio (MMR) also dropped from 871 in 2000 to 673 in 2005 per 100,000 live births, but remained the same in 2011, which is due to the chronically low skill birth coverage and delay in expansion of health centers with emergency obstetric care [10].

Health facility data is important for assessment of progress and performance of national programs; however the quality of the data is affected by a number of limitations, such as completeness, accuracy and external consistency. In particular, the use of such data for trend analysis is also affected by any change over time of the factors affecting the quality of data. However, there was no major systematic change on these factors over the study period, and it is adequate to reliably indicate the trend in service coverage assuming the ‘imperfect’ health facility data is consistent over time. Thus, the observed change in indicators is more likely due to change in health service coverage and health-seeking behaviors brought about by the expansion of services through HEP, although formal systematic evaluation is required to determine the impact of HEP.

## Discussion and evaluation

The nationwide implementation of HEP is progressing in line with targets. The coverage of most preventive health programs such as immunization, antenatal care, family planning and environmental health has improved tremendously since the launch of HEP. However, the performance of the program in improving the coverage of skilled delivery and postnatal care has been limited. Designed with consideration for the social, cultural, political, economic, and health status contexts of the country, the HEP has come to be considered the most important institutional framework for achieving the health MDGs in Ethiopia, but the following challenges may affect its performance.

## Human resource capacity

Despite the gains in physical access to HEP services, the utilization of some services is very low, which could be due to challenges in both supply (poor service quality and service availability) and demand (awareness and cultural barriers) side [33]. Although many aspects of primary healthcare are normally provided by low-level professionals or lay persons with training, it is the quality of training that determines the quality of service and performance of the program [34,35]. The training of HEWs for 1 year on the 17 HEP packages may be inadequate to attain the required knowledge and skills leading to delivery of poor quality services and bypassing of the primary care by users [35,36]. The multitasking by providing 17 service packages by HEWs could affect the availability of services, resulting in inefficiencies of some of the program components [37]. Despite the physical access to health posts, the number of people per HEW (about 2,500 people), which is higher than the widely recommended number of people per community health worker [11,18], could also affect service availability and utilization [5]. The level of awareness, socioeconomic factors, and cultural barriers also contribute considerably to the low service utilization, particularly assisted skilled delivery [33].

### Infrastructure

The investment in critical infrastructure is lagging behind. The health posts require inputs that strengthen the functional system of the services including medical equipments, drugs and supplies, referral systems, standards, guidelines and various health information records [38]. Continuity of care is still a challenge because the target for the number of health centers that provide emergency obstetric care (3,300) has not been achieved; currently, only a third of the 2,660 health centers provide B-EMOC. Increasing access to this service requires significant investment to both upgrade existing health centers and to construct additional ones. The current level of investment is limited due to inadequate total health expenditure, which is currently about US$16 per capita per year [4]. Most of the different sources of cost estimation for the scaling up of specified packages of health interventions indicate a higher cost per person per year than the current health expenditure in the country [18,27,28].

### Management capacity

There is concern about the capacity of the DHOs to manage and provide support to the new health facilities and the large number of new cadres of health workers. HEWs are not receiving quality supervision because HEW supervisors are poorly resourced, leading to dissatisfaction [25,38]. If issues related to salary and benefits, accommodation, working environment, recognition of skills and the availability of career opportunities are not appropriately addressed, they will affect the morale and retention of health personnel in the field, which would negatively affect the performance of the program [25,26,38].

### Monitoring and evaluation

Although the need to base health policies and strategies on sound evidence is increasingly recognized [39], the effort on monitoring and evaluation of the program is limited [38]. Generally, low-income and middle-income countries devote only a small fraction of health expenditure to health research [40], and resources could be wasted in the absence of evidence-based interventions and sustained progress.

The recommendations below are made to address the key challenges in order to achieve national and international (MDG) targets before 2015.

The quality and quantity of human resource capacity should be developed to improve quality of service. There is a need to improve the knowledge and skills of HEWs through targeted training on development of critical skills such as assisted delivery, neonatal and childcare, and identification of danger signs for referral [34,36]. Motivation and satisfaction among health workers is equally important in the provision of quality services [25,41,42], thus, efforts are needed to improve the working and living conditions of HEWs in order to maintain their motivation levels and increase retention [21,43]. Recognition of HEW skills and their valuable contribution should be considered through the award of incentives, salary reviews, accommodation standards, upgrading and refresher training, and promotion based on performance [41]. Increasing the number of HEWs, from the current standard of 2 per kebele of 5,000 people, needs consideration; however it should be based on research evidence as it requires significant investment [18]. Licensure, accreditation and regulation are critical elements to achieve optimal quality standards and assure public safety. Although these issues are considered loosely in the HEP strategy, there is a need to develop and define standard licensure, accreditation and regulation procedures by appropriate legislation at national level. Development of standard HEW time use based on priority interventions and local health context would also contribute to improved service utilization. Provision of the necessary medical equipment, drugs and other supplies is important to increase utilization of services. By improving the quality of services, such measures are important to maximize utilization and ensure efficiency of the HEP [44].

Creation of demand through community mobilization is a key factor to maximize utilization [45]. Improving community participation and acceptance through regular communication to understand and identify their needs is critical for increasing service utilization and continuous improvement of services [30]. In particular, to address the low assisted delivery coverage requires extra efforts; for example, the main focus of the one-to-five women development network should be on promotion of antenatal uptake and ensuring these women deliver their babies at health facilities [33].

However, even if a high coverage of skilled delivery is achieved at the health post level, access to emergency obstetric care is essential to achieve the MDG on maternal mortality [10,13], thus, there is a need to speed up the expansion of referral health centers that provide emergency obstetric care. Moreover, the services at the health post and referral health centers should be linked with a functional referral system (supported by means of transportation) to ensure continuum of care [10,45].

Strengthening the DHOs in management, technical and logistic support and supervision is key for successful implementation and provision of quality care [15,24]. This would require training of district health managers in effective leadership and management, drug supply management, monitoring and use of data for decision making; and strengthening the skills of supervisors in effective supervision techniques.

Research is critical to bridge the implementation gap through evaluating activities and systems [46,47]. The implementation of HEP should be accompanied by monitoring and evaluation studies to demonstrate that the goals and objectives are being achieved and to document factors that affect the success of the program focusing on areas of relevance to policymakers [39,48]. Implementation research on the effectiveness of the various service delivery models is critical to policymakers [30,46]. Identification of barriers (both supply and demand side) that prevent people from utilizing services that are available within their villages is important to deliver the service in ways acceptable to the communities [21]. Assessment of the technical capacity, time use, workload, the perception and satisfaction of HEWs, retention strategies as well as the health facility performance are critical to improve the quality of service [7,25,26,49].

## Conclusions

The Ethiopian approach of revitalization of primary healthcare through innovative and locally appropriate and acceptable strategies can provide important lessons to other countries to emulate the strategy and reform it under their local conditions, recognizing that a ‘one-size-fits-all’ strategy to address the poor health status of all developing countries will not work as countries are very diverse in terms of culture, political circumstances, administrative capacities, language, national economic resources, and historical development of health sector. However, the reality of implementation of such health reform depends on the relative presence of political interest, will and capacity to implement reform. It is hoped that the innovative approaches and strategies described in this paper will aid in that process.

## Abbreviations

ANC: Antenatal care; B-EMOC: Basic emergency obstetric care; CHW: Community health workers; C-EMOC: Comprehensive emergency obstetric care; FMOH: Federal ministry of health; FP: Family planning; HSDP: Health sector development program; HEP: Health extension program; HEW: Health extension worker; HMIS: Health management information system; MDG: Millennium development goal; PHCU: Primary health care unit; PNC: Postnatal care; RHB: Regional health bureau; DHO: District health office.

## Competing interests

The authors declare that they have no competing interests.

## Authors’ contributions

Both authors participated in data collection, analysis, and drafted the manuscript. Both authors revised and approved the final manuscript.
